# Special Issue on Ontologies and Data Management: Part I

**DOI:** 10.1007/s13218-020-00682-7

**Published:** 2020-09-16

**Authors:** Thomas Schneider, Mantas Šimkus

**Affiliations:** 1 University of Bremen, Bremen, Germany; 2 TU Wien, Wien, Austria

## Introduction

Modern information systems rely on a large amount of data that is often unstructured, heterogeneous, and/or incomplete. In order to align and complete data, these systems often use ontologies for representing taxonomies and background knowledge. Ontologies have been used in the Knowledge Representation (KR) subfield of Artificial Intelligence (AI) since the 1970s. They have various applications in (bio)medicine, the life sciences, linguistics, the geo-sciences, and the semantic web. Systems that use ontologies do not only access the represented knowledge, but also draw inferences, a process known as automated reasoning. Among existing ontology languages, the family of *description logics (DLs)* plays an important role because DLs usually provide a good balance between expressive power and decidability/complexity of the various reasoning tasks, also and especially those tasks relevant for data access. The standardized ontology language OWL 2 recommended by the World Wide Web Consortium (W3C) is based on DLs. However, there are further suitable ontology languages, such as existential rules.

The title of this double special issue, *Ontologies and Data Management*, refers to a wide research landscape that stretches over various topics from knowledge representation and reasoning. This landscape includes theory and applications, and connects with the database “world”. The research areas covered in both parts of this special issue include ontologies, ontology languages, modelling support, reasoning systems, reasoning problems and ontology-based data access, and these research areas are highly active. This special issue gives evidence to this, as it presents a broad collection of current work on topics from these areas. Most of the contributions are related to ontologies written in OWL and research on DLs. The vastness of the research landscape is reflected by the diversity of contributions in this and the next part of the special issue.

A lot of people have put very significant efforts into making this special issue a reality, and we sincerely thank them. Everybody involved was and still is significantly affected by the COVID-19 crisis that hit the world in the beginning of 2020, which makes the efforts of the authors, the reviewers and all the supporting staff even more precious. We would like to thank all the authors for submitting high quality papers, and also for carefully revising them following suggestions by the reviewers. We also sincerely thank the reviewers for doing an excellent job and providing valuable feedback. Thanks to the authors and the reviewers this special issue is a collection of top quality works! The editors would also like to thank Anni-Yasmin Turhan and the Springer team for helping with all aspects of preparing this special issue. The work of the second guest editor was supported by the Vienna Business Agency, and the Austrian Science Fund (FWF) projects P30360 and P30873.

## About this Special Issue

This special issue is divided into two parts (Part I and Part II). In this editorial, we concentrate on the contributions of the first part. The second part will be published in December 2020 (as Issue 4 of Volume 34). In particular, the contributions in Part II will be mostly focused on *inconsistency management*, *non-monotonic reasoning* and the involvement of *rules* in the context of ontologies. The content of Part I is listed in the following section. We next provide a brief overview of those contributions, which are divided into technical contributions [T1–T5], system descriptions [S1–S4], PhD thesis abstracts [A1–A4], project reports [P1–P2], and include an interview [I1] with Uli Sattler, a leading researcher in Description Logics.

The first technical contribution [T1] by Baader and Théron presents results on the decidability of the DL $\mathcal {FL}_0$ extended with *role-value maps*, which constitute a concept constructor that is very useful for knowledge representation yet very challenging computationally. The contribution [T2] by Bonatti et al. shows how OWL 2 can be used for improving management of personal data in the context of the European General Data Protection Regulation (GDPR). In her contribution [T3], Ozaki provides an overview of several approaches to constructing high quality DL ontologies from (possibly very noisy) data, a challenging area that uses diverse techniques from machine learning, probabilistic reasoning, classic symbolic reasoning and others. The survey [T4] provides a brief overview on DL-based ontologies and data management. The paper [T5] by Toman and Weddell shows how reasoning in *feature-based* DLs—a family of DLs suitable for expressing traditional relational database constraints—can be used in the context of database query optimization.

The system description [S1] by Braun et al. presents a new web-based visual tool to support ontology development in a collaborative environment. Fillottrani et al. describe in [S2] a system for linking data to ontological knowledge in the recently introduced KnowID architecture. Koopmann presents in [S3] a tool for forgetting and uniform interpolation in expressive DLs, which are reasoning tasks playing a crucial role in enabling ontology reuse, aiding ontology analysis and privacy. The system description [S4] by Manthey et al. presents a system for *axiom pinpointing* (a reasoning task for explaining inferences from ontologies) in the lightweight DL $\mathcal {EL}^{+}$, which reuses existing highly efficient SAT solving technologies.

The PhD thesis abstract [A1] describes the contributions of Bajraktari in the area of query answering in expressive DLs, with the special focus on exploiting the structure of data to improve efficiency. Kriegel describes in [A2] his PhD research into how methods from the area of *Formal Concept Analysis* can be used to support the construction DL ontologies from data. This work is related to the problem of learning DL ontologies discussed in [T3], which again indicates the existing interest in this area. The abstract [A3] by Marantidis describes his PhD research into (approximate) unification in the DL $\mathcal {FL}_0$, which is a reasoning task useful for the development and maintenance of ontologies. The PhD thesis abstract [A4] by Nuradiansyah describes his research into the use of DL ontologies for privacy management. Together with [T2] this is a second contribution on privacy, which shows the current importance of this area.

The project report [P1] by Homola et al. describes the efforts to obtain *higher-order* DLs that could become a basis for a higher-order ontology language for the Semantic Web. Such extensions are highly desirable because they drastically improve the expressiveness of ontology languages by, e.g., blurring the distinction between classes and individuals. Finally, the report [P2] by Horsch et al. discusses the use of ontologies for data management in the Horizon 2020 project *Virtual Materials Marketplace (VIMMP)*.

The interview [I1] with Uli Sattler gives an insight into her background, her research interests and outcomes, and her personal views on research in description logic and related areas, on the OWL standard, and on possible future developments of knowledge representation and reasoning.

## Content

### Technical Contributions

1. *Role-Value Maps and General Concept Inclusions in the Minimal Description Logic with Value Restrictions or Revisiting Old Skeletons in the DL Cupboard*
     Franz Baader and Clément Théron
2. *Machine Understandable Policies and GDPR Compliance Checking*
      Piero A. Bonatti, Sabrina Kirrane, Iliana M. Petrova and Luigi Sauro
3. *Learning Description Logic Ontologies Five Approaches. Where Do They Stand?*
     Ana Ozaki
4. *Ontologies and Data Management: a Brief Survey*
     Thomas Schneider and Mantas Šimkus
5. *Using Feature-Based Description Logics to avoid Duplicate Elimination in Object-Relational Query Languages*
     David Toman and Grant Weddell

### System Descriptions

1. *crowd: A Visual Tool for Involving Stakeholders into Ontology Engineering Tasks*
     Germán Braun, Christian Gimenez, Laura Cecchi and Pablo Fillottrani
2. *Connecting knowledge to data through transformations in KnowID: system description*
     Pablo R. Fillottrani, Stephan Jamieson and C. Maria Keet
3. *LETHE: Forgetting and Uniform Interpolation for Expressive Description Logics*
     Patrick Koopmann
4. *SATPin: Axiom Pinpointing for Lightweight Description Logics Through Incremental SAT*
     Norbert Manthey, Rafael Peñaloza and Sebastian Rudolph

### Thesis Abstracts

1. *Querying Rich Ontologies by Exploiting the Structure of Data*
     Labinot Bajraktari
2. *Constructing and Extending Description Logic Ontologies using Methods of Formal Concept Analysis: A Dissertation Summary*
     Francesco Kriegel
3. *Quantitative Variants of Language Equations and their Applications to Description Logics*
     Pavlos Marantidis
4. *Reasoning in Description Logic Ontologies for Privacy Management*
     Adrian Nuradiansyah

### Project Reports

1. *Towards higher-order OWL*
     Martin Homola, Ján Kľuka, Petra Hozzová, Vojtěch Svátek and Miroslav Vacura
2. *Ontologies for the Virtual Materials Marketplace*
     Martin Thomas Horsch, Silvia Chiacchiera, Michael A. Seaton, Ilian T. Todorov, Karel Šindelka, Martin Lísal, Barbara Andreon, Esteban Bayro Kaiser, Gabriele Mogni, Gerhard Goldbeck, Ralf Kunze, Georg Summer, Andreas Fiseni, Hauke Brüning, Peter Schiffels and Welchy Leite Cavalcanti

### Interviews

1. Interview with Uli Sattler

## Service

We next provide a list of selected venues, in which research related to the use of ontologies in data management, especially involving automated reasoning, is disseminated.

### Conferences, Workshops, and Summer Schools

- AAAI Conference on Artificial Intelligence (AAAI)
- Alberto Mendelzon International Workshop on Foundations of Data Management (AMW)
- International Workshop on the Resurgence of Datalog in Academia and Industry (Datalog 2.0)
- International Workshop on Description Logics (DL)
- European Conference on Artificial Intelligence (ECAI)
- International Joint Conference on Artificial Intelligence (IJCAI)
- International Conference on Database Theory (ICDT)
- International Semantic Web Conference (ISWC)
- European Conference on Logics in Artificial Intelligence (JELIA)
- International Conference on Principles of Knowledge Representation and Reasoning (KR)
- ACM–IEEE Symposium on Logic in Computer Science (LICS)
- International Conference on Principles of Database Systems (PODS)
- International Joint Conference on Rules and Reasoning (RuleML+RR)
- Reasoning Web Summer School (RW)

### Journals

- Journal of Artificial Intelligence Research
- Artificial Intelligence
- ACM Transactions on Computational Logic

